# A reproducible workflow for diagnosing YOLO26-to-NCNN deployment inconsistencies on resource-constrained Android devices

**DOI:** 10.1016/j.mex.2026.104064

**Published:** 2026-07-25

**Authors:** Jun Ma, Liangying Xu, Xuanqiong Li, Yanlong Liang

**Affiliations:** a Department of Surveying and Mapping, Sichuan University of Architectural Technology, Deyang, 618000, China; b Department of Science and Technology, Chengdu Qinggong Polytechnic University, Chengdu, 611731, China

**Keywords:** Android deployment, NCNN, YOLO26, Deployment consistency, Model conversion, Reproducible workflow

## Abstract

Edge devices increasingly require reliable conversion of desktop YOLO26 models into Android runtimes. This article presents a reproducible YOLO26-to-NCNN diagnosis workflow for resource-constrained Android phones, demonstrated with a 10-class SafeHat PPE task. The method links export, Android asset replacement, Java-JNI-C++ inference, logging, five-stage fault localization, and frozen-image regression. It targets silent deployment errors such as high empty-scene scores, fixed 0.5 confidence, missing geometry, and label disorder. Validation across detection, segmentation, pose, classification, and oriented-box assets shows that letterbox, activation, coordinate-semantic, and layout faults can be localized and repaired, with CPU-path evidence from two Android devices.

- Reproducible diagnosis workflow for YOLO26-to-NCNN Android deployment inconsistencies.

- Five-stage fault localization from Android symptoms to verified repair artifacts.

- Validation package with scripts, frozen images, logs, and CPU-device evidence.

## Graphical abstract

**Figure:**
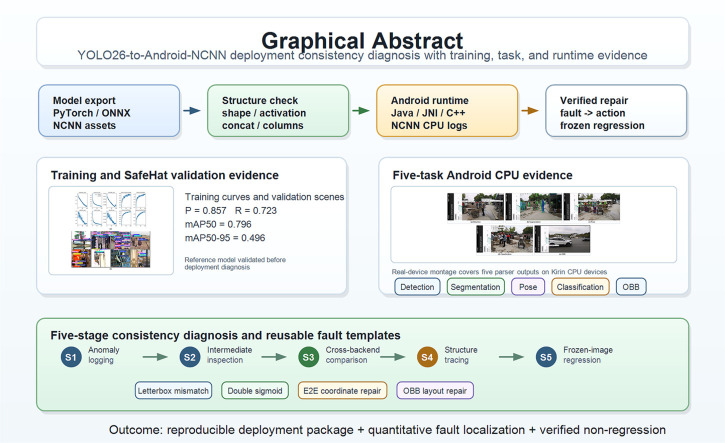

Specifications table

**Table:** 

**Subject area**	Computer Science
**More specific subject area**	Computer vision; mobile deep-learning inference; Android deployment; reproducible model-conversion diagnostics
**Name of your method**	YOLO26-to-NCNN deployment consistency diagnosis workflow
**Name and reference of original method**	YOLO-family object-detection deployment through NCNN Android runtime
**Resource availability**	https://github.com/majun2019/ncnn-android-yolo26lt; https://doi.org/10.5281/zenodo.20137180

## Background

Mobile visual inference often depends on converting a desktop-trained YOLO26 model into an Android runtime. Model preparation and interchange commonly use Ultralytics export and ONNX-format conversion checks [1,2]. On Android, exported models can be executed by mobile inference runtimes such as NCNN or TensorFlow Lite [3,4]. Alternative runtimes, including ONNX Runtime Mobile and MNN, are used when operator support, deployment tooling, or platform constraints differ [5,6].

The deployment side must also follow changes in model-output semantics. Early YOLO-style dense predictions and newer end-to-end detection structures require different parser assumptions [7,8]. Contemporary real-time detectors such as YOLOX and YOLOv7 further illustrate the pace of detector architecture change [9,10], while DETR comparisons show continued diversification of detection outputs [11]. Segmentation and oriented bounding-box tasks add mask, angle, or geometry-specific outputs that require task-aware parsing [12,13]. Recent Android object-detection applications confirm the practical demand for YOLO-family models on mobile devices [14,15].

Android integration adds another source of inconsistency. Native interfaces and camera pipelines are usually built through Android NDK/JNI and CameraX [16,17]. OpenCV and Vulkan provide common image-processing and acceleration foundations [18,19], and PNNX provides a PyTorch-to-NCNN conversion reference for graph inspection [20]. This chain is practical for edge deployment, but it also creates deployment-consistency risks: a model that behaves correctly on the desktop may produce different semantics after export and Android-side post-processing.

The most damaging errors are silent. The application may keep running while empty scenes receive high confidence, confidence values stay near 0.5, pose skeletons disappear, oriented boxes shift outside the image, or class labels become systematically wrong. Such symptoms cannot be diagnosed reliably from a single screenshot because the cause may lie in preprocessing, graph conversion, tensor traversal, parser layout, or UI score mapping.

This article treats those errors as a reproducibility problem. It provides a compact YOLO26-to-Android-NCNN workflow for resource-constrained CPU devices, using a SafeHat PPE task as the main demonstration while retaining transfer checks for detection, segmentation, pose, classification, and oriented bounding-box assets. The contribution is a reproducible deployment protocol, a five-stage diagnosis workflow, and a validation package built from project scripts, Android logs, frozen images, and two low-end phones. Rather than proposing a new primitive, the method provides a practically reusable diagnosis loop that systematically traces Android symptoms to verified regression evidence.

## Method overview

### Problem definition

Current toolchains verify model conversion but cannot systematically trace runtime symptoms across execution stages. The workflow diagnoses inconsistency between a desktop-side YOLO26 model and an Android-side NCNN application [1,3]. It assumes a trained YOLO26 model, an NCNN export path, an Android application that loads param/bin files, and at least one reference input that can be run in more than one backend [2,5].

The workflow treats the Android inference path as a sequence of observable transformations, from conversion artifacts to native execution and rendering [16,17]:

training model -> exported graph -> NCNN param/bin -> Android asset -> JNI/native inference -> task parser -> post-processing -> rendered output/log.

**Fig. 1:**
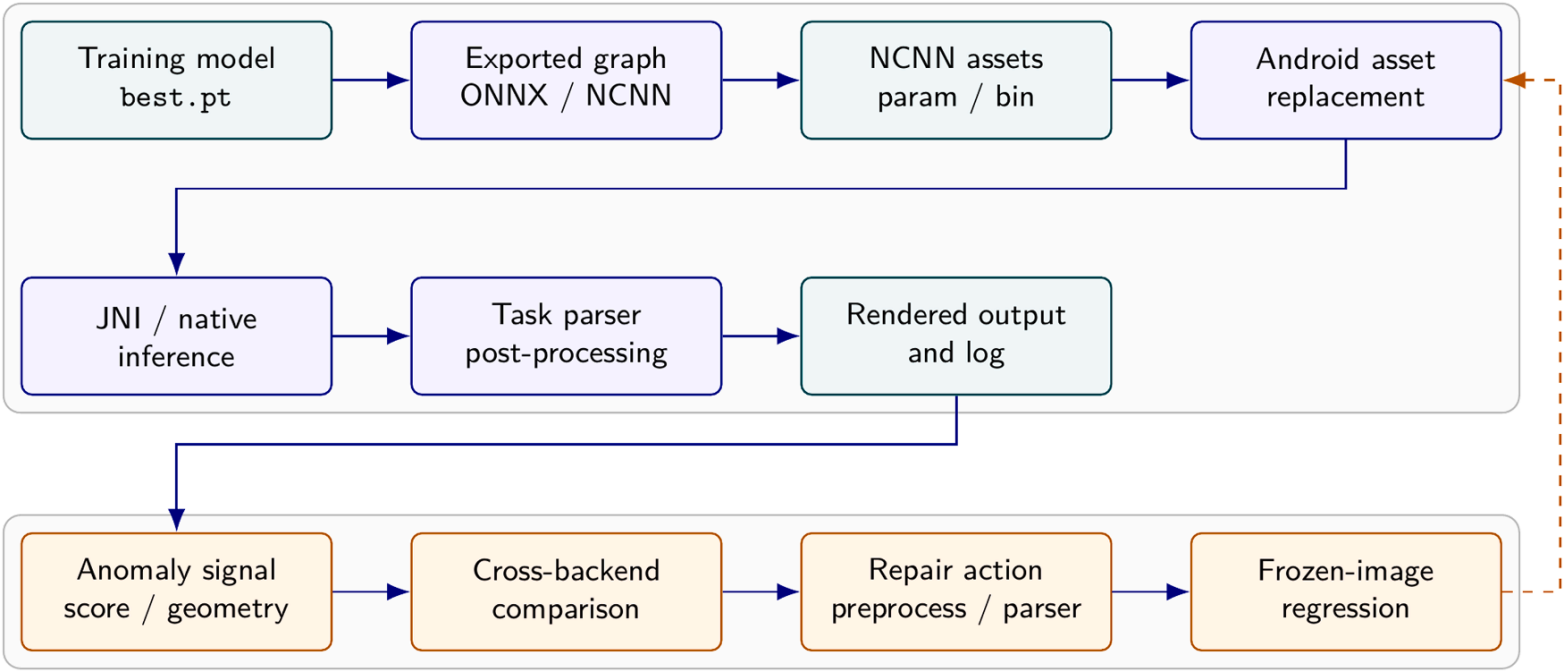
Model export and on-device validation closed loop.

The method receives Android symptoms and returns a fault category, a suspected root cause, a repair action, and a regression artifact. Fig. 1.

### Inputs, outputs, and artifacts

The workflow is designed to operate with files that are already present in the project. Table 1 summarizes the inputs and reusable artifacts.

**Table 1:** Method inputs, tools, and artifacts.

Object	Project example	Role in the method
PyTorch model	best.pt, yolo26n.pt	Desktop-side reference or source model
Dataset definition	scripts/safehat.yaml	Class names, task semantics, input size
NCNN assets	app/src/main/assets/*.ncnn.param, *.ncnn.bin	Android-loadable model files
Android native parser	app/src/main/jni/yolo26_*.cpp	Output traversal, decoding, task-specific post-processing
Java/JNI interface	YOLO26Ncnn.java, MainActivity.java	Task switching, threshold update, model loading
Diagnosis scripts	scripts/diagnose_model.py, trace_conversion_pipeline.py, conversion_detail_check.py, verify_ncnn_output.py	Backend comparison and structure tracing
Regression scripts	test_letterbox_hypothesis.py, test_yolo26_e2e.py, sensitivity_sweep.py	Repair validation and frozen-image sweep
Runtime logs	logcat.txt, newtest.log, YOLO26BENCH lines	Latency, loading, object count, diagnostic scores
Frozen images	data/testdata/target_present, empty_background, hard_negative	Re-checking and parameter sensitivity demonstration

### Five-stage diagnosis logic

**Fig. 2:**
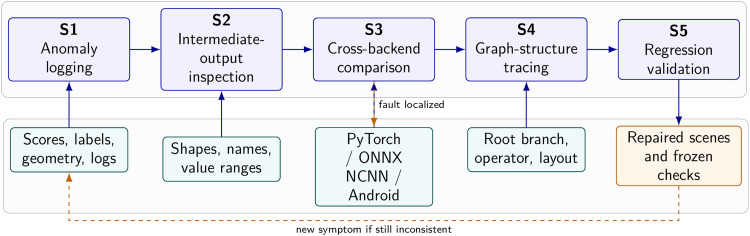
Five-stage fault diagnosis workflow for android deployment.

The method is organized as five decision stages. Not every case requires all stages; for example, cross-backend comparison may directly isolate a preprocessing fault. Regression validation remains mandatory after any repair. Table 2.Fig. 2.

**Table 2:** Five-stage diagnosis workflow.

Stage	Name	Input	Operation	Exit criterion	Project tools
S1	Anomaly logging	Android logs, screenshots, rendered output	Record abnormal scores, fixed confidence values, missing geometry, label disorder, or loading errors	The symptom is reproducible and numerically described	logcat, diagnose_background_scores.py, collect_latency.py
S2	Intermediate-output inspection	NCNN param/bin, tensor shapes, parser assumptions	Inspect output names, output widths/heights, class count, and tensor value ranges	The deviation is localized to a tensor shape, numerical range, or parser expectation	verify_ncnn_output.py, diagnose_model.py
S3	Cross-backend comparison	Same image in PyTorch, ONNX, Python-NCNN, Android-NCNN	Compare output distributions and rendered behavior across backends	The fault is assigned to export/conversion or Android-side implementation	trace_conversion_pipeline.py, diagnose_model.py
S4	Graph-structure tracing	NCNN param graph and native parser code	Trace Sigmoid nodes, concat chains, output-column order, and coordinate semantics	The root branch, operator, or column assumption is identified	conversion_detail_check.py, parser inspection
S5	Regression validation	Repaired app and frozen scenes	Re-run affected scenes and previously passing scenes	The abnormal diagnostic signal is removed and no known passing case regresses	test_letterbox_hypothesis.py, test_yolo26_e2e.py, sensitivity_sweep.py

## Method details

### Software assets and environment

The implementation uses a Java-JNI-C++-NCNN Android application. Direct control over preprocessing, parser code, and logging is required because many faults are introduced after the exported model is already valid. The demonstration combines Ultralytics export [1], ONNX-related checks [2], NCNN mobile inference [3], OpenCV-Mobile image operations [18], Android NDK/JNI components [16], and a Vulkan-capable NCNN package while reporting CPU-path measurements [19].

The SafeHat demonstration uses 10 classes: Hardhat, Mask, No-Hardhat, No-Mask, No-Safety Vest, Person, Safety Cone, Safety Vest, Machinery, and Vehicle. The project contains 2685 training images, 114 validation images, and 48 frozen method-validation images. Training metrics are used only to confirm that the demonstration model is usable before deployment diagnosis.

### YOLO-to-NCNN export and asset deployment protocol

The protocol converts a trained model into Android assets and checks whether output tensors still match the native parser. The reproducible steps are: load or fine-tune a SafeHat model from safehat.yaml, export best.pt to NCNN param/bin files, inspect graph structure and output dimensions, replace assets under app/src/main/assets/, and run on-device logging with reference scenes. The corresponding failure signals include missing class names, export errors, unsupported operators, unexpected output dimensions, loadModel failures, high background scores, fixed confidence values, missing geometry, and label disorder.

The detection demonstration uses yolo26n_safehat.ncnn.param/bin as the preferred semantic asset name. A historical compatible alias, yolo26n_e2e, is also present. In the SafeHat case, the detection output is treated as an O2M tensor with 8400 proposals and 10 classes rather than as a fully decoded E2E 300×6 tensor.

### Android-NCNN inference and logging path

The Android path has four functional layers: Java interaction for task selection and thresholds, JNI transfer for task IDs and native lifecycles, C++ inference for model loading and task parsing, and NCNN/OpenCV/NDK runtime support. The same application exercises detection, segmentation, pose estimation, classification, and oriented bounding-box assets. For each task, the diagnosis checks input size, output shape, class count, decoded-coordinate semantics, and parser column layout.

The YOLO26BENCH log tag records model loading, task switching, per-frame inference time, object count, and summary statistics. Additional native logs record raw and calibrated detection scores. These logs provide the S1 anomaly baseline and the S5 regression artifact.

### Preprocessing and post-processing rules

The workflow treats preprocessing as a first-class consistency check. For the four 640×640 vision tasks, the Android path uses letterbox resizing with padding value 114. Let the original width and height be W and H, and let the target side length be S. The scale factor and resized size are

$r=min\left(S/W,\;S/H\right),\;W^{\prime }=\lfloor rW\rfloor ,\;H^{\prime }=\;\lfloor rH\rfloor .$

The padding values are

$p_{x}\;=\;\lfloor \left(S-W^{\prime }\right)/2\rfloor ,\;p_{y}=\;\lfloor \left(S-H^{\prime }\right)/2\rfloor .$

When a box is mapped back to the original image,

$x=\left(\hat{x}-p_{x}\right)/r,\;y=\left(\hat{y}-p_{y}\right)/r,\;w=\hat{w}/r,\;h=\hat{h}/r.$

The repair rule is to pad the input to a strict target_size x target_size tensor, not merely to a stride-aligned rectangle. The latter can produce a 640×384 tensor for a 1920×1080 input and changes the input distribution seen by the model.

For the SafeHat detection demonstration, the device reports a diagnostic display score.

${\tilde{p}}_{dev}\;=\;\sigma \left(\frac{\log(p/(1-p))}{T_{dev}}\right),\;T_{dev}\;=3.$ Where p is the raw model probability and σ is the sigmoid function. This transformation is used for diagnostic display and threshold readability; it is not presented as a detector-accuracy improvement. The offline parameter sweep uses a two-stage display-compression variant.

${\tilde{p}}_{T,\gamma }\;=\;{\left[\sigma (\frac{\log(p/(1-p))}{T})\right]}^{\gamma }.$

The SafeHat post-processing path also includes per-class Top-K candidate retention, per-class NMS, mutually exclusive suppression for conflicting class pairs such as Hardhat and No-Hardhat, person-dependent PPE association, and PPE box locking. These mechanisms are kept in the method description because they create observable diagnostic signals during regression, but their parameter sweep is interpreted as a protocol demonstration rather than as a universal parameter recommendation.

### Fault-specific repair templates

The workflow converts recurrent faults into four repair templates. A preprocessing mismatch is suspected when empty scenes produce many high-confidence detections; the repair emits a strict 640×640 letterbox tensor with padding value 114. A redundant activation is suspected when confidence values collapse near 0.5; graph tracing checks whether sigmoid is already present, and the parser-side duplicate is removed. A coordinate semantic mismatch is suspected when pose skeletons or OBB geometry disappear; decoded E2E outputs are then used directly. An OBB layout mismatch is suspected when labels, scores, and directions are systematically wrong; concat-chain tracing verifies the actual [bbox, classes_sigmoided, angle_raw] layout and corrects parser indices.

## Method validation

### Demonstration case and available data

The demonstration case is SafeHat PPE detection on Android-NCNN. It provides the complete closed loop: hard-negative mining, fine-tuning, NCNN export, Android asset replacement, on-device execution, fault repair, and regression validation. Segmentation, pose, classification, and OBB assets are used to check whether the same application path handles heterogeneous output formats.

**Fig. 3:**
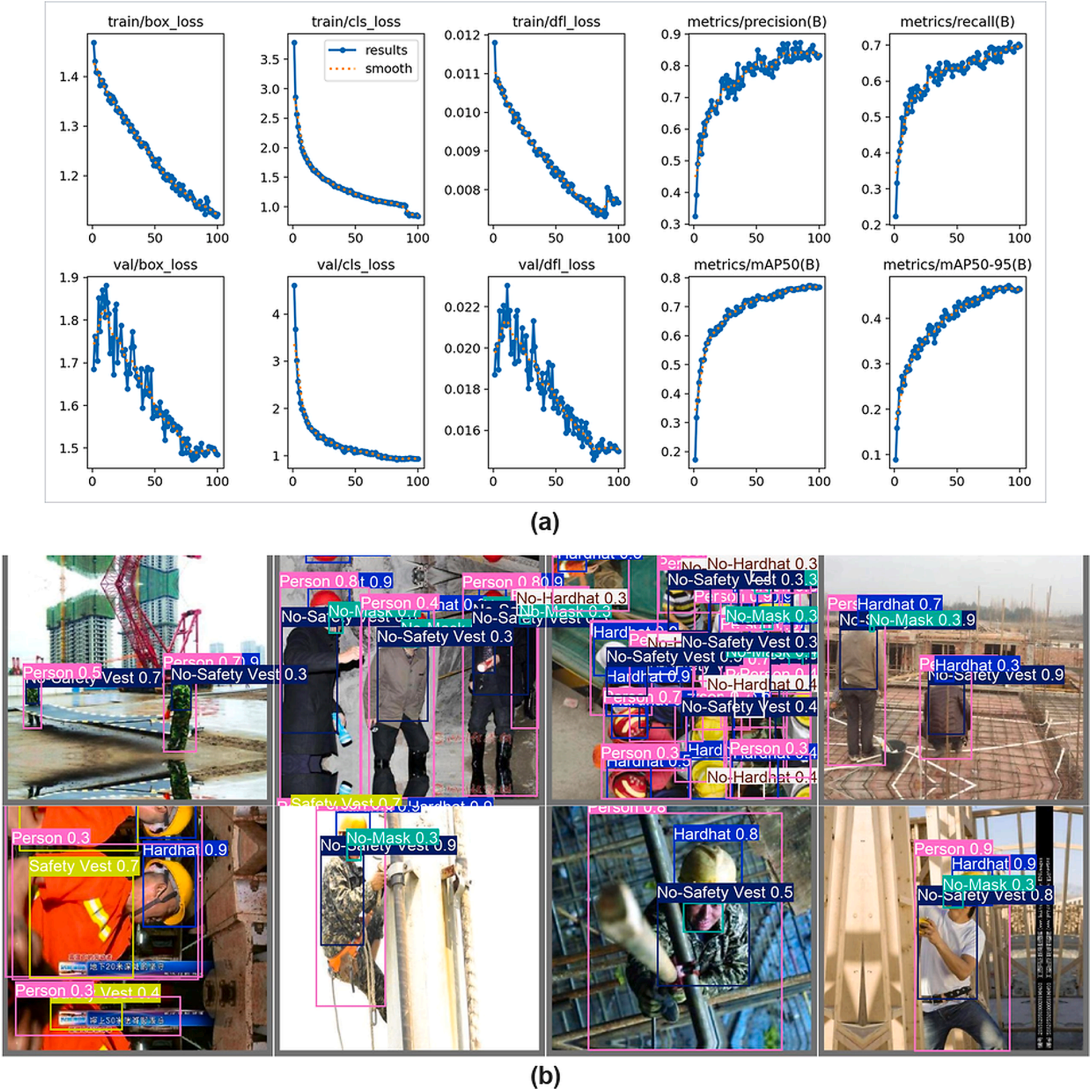
SafeHat training curves and validation examples used as the pre-deployment sanity check.

The SafeHat demonstration contains 10 classes: Hardhat, Mask, No-Hardhat, No-Mask, No-Safety Vest, Person, Safety Cone, Safety Vest, Machinery, and Vehicle. Training-side curves and validation examples are used only as a pre-deployment sanity check. Fig. 3 shows this evidence.

### Validation on four representative fault modes

Four representative faults validate the workflow. The preprocessing fault was localized to stride-aligned 640×384 padding for a non-square camera frame; enforcing a strict 640×640 letterbox tensor reduced empty-scene median scores from 0.84–0.96 to below 0.0001. The redundant-activation fault was localized by graph tracing: NCNN already contained sigmoid, while the C++ parser applied sigmoid again. Removing the duplicate restored scene-dependent probabilities with p_{50} ≈ 0.34 and p_{95} ≈ 0.74. Coordinate-semantic repair restored pose and OBB geometry by using decoded E2E outputs directly. OBB-layout tracing corrected the parser assumption from [bbox, angle, classes] to [bbox, classes_sigmoided, angle_raw], restoring labels, confidence values, and directions.

### Reproducibility artifacts and regression checklist

Method reuse depends on whether another user can reproduce the diagnosis steps. The reusable package contains Android source under app/src/main/, NCNN assets under app/src/main/assets/, training/export scripts, tensor and graph diagnosis scripts, letterbox and E2E regression scripts, frozen images under data/testdata/, runtime logs, and the public repository/archive.

For a new deployment, the minimal regression checklist is:

1. Confirm that the Android app loads the expected param/bin file pair.
2. Confirm that task-specific output shape and class count match the parser.
3. Run an empty-background input and record background score quantiles.
4. Run at least one target-present input and confirm a plausible raw-score range.
5. Compare the same input between desktop reference and Android-side NCNN if a symptom appears.
6. Inspect graph-level activations and output-column layout when value-range comparison is insufficient.
7. Re-run the previously failing input and at least one previously passing input after repair.

### Parameter sensitivity demonstration

The frozen sweep is a reproducibility check rather than a universal parameter study. The script scripts/sensitivity_sweep.py evaluates 48 cached outputs: 18 target-present, 15 empty-background, and 15 hard-negative images. Across all settings, frac_gt090 remained 0.000 because the display-compression transform suppresses over-confident display values. Representative settings show that T = 1 gives the widest target-present display range [0.330, 0.586], while T = 6 compresses it to [0.330, 0.371]. Changing γ from 1.0 to 2.0 shifts the range from [0.500, 0.538] to [0.250, 0.290]. Per-class Top-K controls candidate retention: K_c = 20, 50, and 100 produce mean empty-scene counts of 62.6, 131.3, and 239.9.

### Runtime feasibility on AndroidCPU devices

**Fig. 4:**
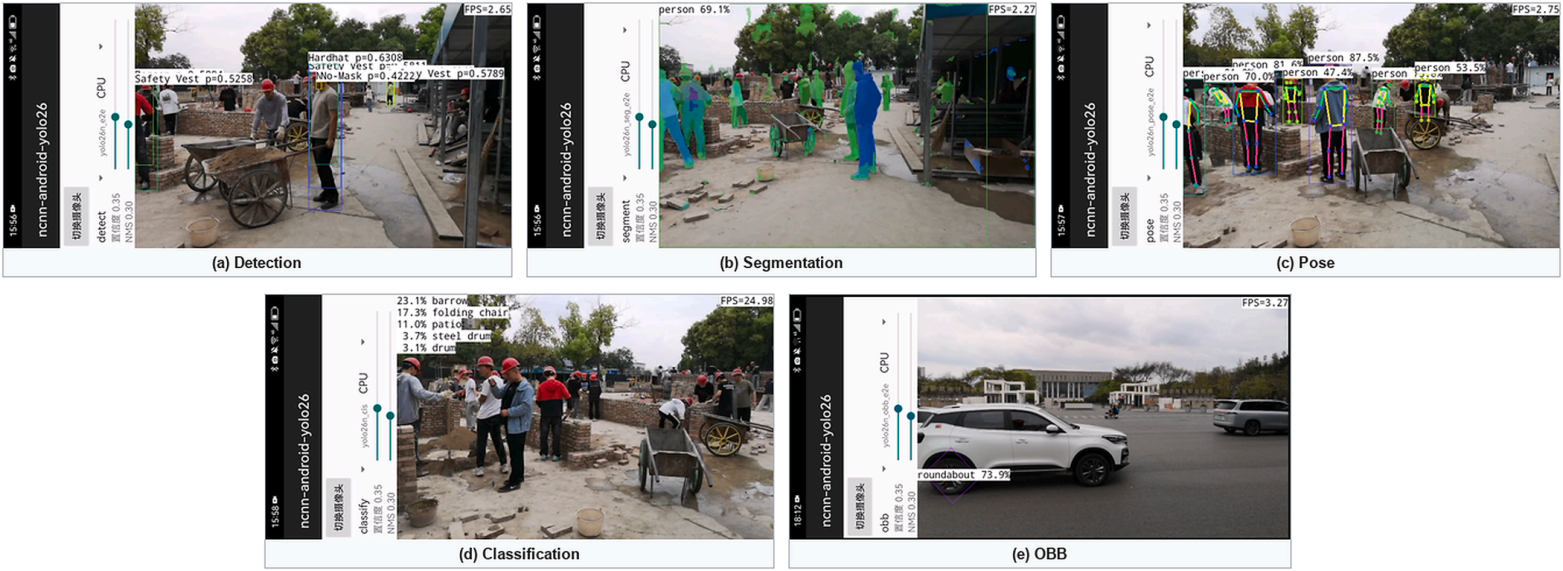
Five-task Android-NCNN runtime montage on CPU devices, covering detection, segmentation, pose estimation, classification, and oriented bounding-box tasks.

Runtime logs verify that the validation package can be exercised on real Android CPU devices. Fig. 4 shows the five-task on-device montage, and Table 3 summarizes per-task latency from two Kirin phones. The numbers indicate validation scale rather than a universal Android benchmark.

**Table 3:** CPU-path runtime evidence from real Android devices.

Task	Kirin 970 mean/FPS	Kirin 659 mean/FPS	Input and note
Detection	321.3 ms / 3.1 FPS	621.5 ms / 1.6 FPS	640×640 letterbox; SafeHat asset
Segmentation	430.5 ms / 2.3 FPS	829.8 ms / 1.2 FPS	640×640 letterbox; mask output
Pose estimation	369.4 ms / 2.7 FPS	740.2 ms / 1.4 FPS	640×640 letterbox; 17-keypoint output
Classification	33.6 ms / 29.8 FPS	70.8 ms / 14.1 FPS	224×224 resize; Top-5 output
OBB	336.1 ms / 3.0 FPS	640.6 ms / 1.6 FPS	640×640 letterbox; oriented-box output

For the Kirin 970 rows, means are derived from frame-level YOLO26BENCH FRAME logs after warm-up removal, except OBB, where the mean uses the 120-frame summary. The Kirin 659 rows use available SUMMARY values from the supplementary session (newtest.log).

## Summary of validated outcomes

This article presents a reproducible YOLO26-to-Android-NCNN deployment and diagnosis method. It links model preparation, NCNN conversion, Android asset replacement, Java-JNI-C++ inference, on-device logging, and frozen-image regression into a protocol that can be repeated with project scripts and Android logs.

The SafeHat demonstration validated four deployment faults: letterbox mismatch, redundant sigmoid, coordinate-semantic mismatch, and OBB output-layout mismatch. The repairs reduced empty-scene scores, restored scene-dependent probabilities, recovered pose and OBB geometry, and corrected label and angle interpretation. The 48-image sweep and two-device CPU logs further show that the method records post-processing and runtime evidence without converting the paper into a detector benchmark.

The contribution is a reproducible deployment protocol, a five-stage diagnosis workflow, and a compact validation package for Android-NCNN deployment of YOLO-based visual inference models.

## Limitations

The workflow is reusable because its decision points are not specific to SafeHat PPE monitoring. Letterbox mismatch, redundant activation, decoded-coordinate misinterpretation, and output-layout mismatch can occur in many YOLO-to-NCNN Android deployments. Each repair is tied to a diagnostic signal and a regression artifact rather than to visual inspection alone.

The method has four boundaries. The most complete quantitative validation is concentrated in the SafeHat detection case; the other task assets mainly test heterogeneous output formats and runtime switching. The 48-image sweep is a frozen protocol demonstration rather than a large benchmark. The latency values come from two Android phones on the NCNN CPU fp32 path and do not compare alternative runtimes or quantized variants. Finally, the workflow benefits from a desktop-side or Python-side reference; without one, tensor inspection and graph tracing carry more of the diagnosis burden.

## Ethics statements

This study used public datasets and project-level demonstration images. It did not involve human-subject intervention, biometric identification, or the collection of new personal data. The Android device experiments measured software behavior and inference latency only.

## CRediT author statement

**Jun Ma:** Conceptualization, Investigation, Supervision, Data curation, Methodology, Validation, Writing – original draft. **Liangying Xu:** Data curation, Methodology, Writing – review & editing. **Xuanqiong Li:** Writing – review & editing. **Yanlong Liang:** Data curation, Methodology.

## Funding

Research on a Smart Campus Management Model Based on Mixed Reality Technology and Spatial Databases of SCAC.

Supplementary material and/or additional information [OPTIONAL]

The deployment framework code is publicly available at https://github.com/majun2019/ncnn-android-yolo26lt (https://github.com/majun2019/ncnn-android-yolo26lt) and is archived on Zenodo with DOI https://doi.org/10.5281/zenodo.20137180 (https://doi.org/10.5281/zenodo.20137180).

The public repository and Zenodo archive contain the Android application source, NCNN assets, scripts, frozen validation images, runtime logs, and reproducibility artifacts referenced in the manuscript.

## Declaration of competing interest

The authors declare that they have no known competing financial interests or personal relationships that could have appeared to influence the work reported in this paper.

## Data Availability

Data/code are available athttps://github.com/majun2019/ncnn-android-yolo26lt
